# In Situ Time-Based Sensor for Process Identification Using Amplified Back-End-of-Line Resistance and Capacitance

**DOI:** 10.3390/s25113255

**Published:** 2025-05-22

**Authors:** Jen-Chieh Hsueh, Mike Kines, Yousri Ahmed Tantawy, Dale Shane Smith, Jamin McCue, Brian Dupaix, Vipul J. Patel, Waleed Khalil

**Affiliations:** 1 Electroscience Laboratory, Ohio State University, Columbus, OH 43212, USA; hsueh.38@osu.edu (J.-C.H.); smith.2341@osu.edu (D.S.S.); khalil.18@osu.edu (W.K.); 2 U.S. Air Force Research Laboratory, Dayton, OH 45433, USA; jamin.mccue.1@us.af.mil (J.M.); brian.dupaix.1@us.af.mil (B.D.); 3 Ansys, Inc., Exton, PA 19341, USA; vipul.patel@ansys.com

**Keywords:** process attestation, back-end-of-line (BEOL), die authentication, RC sensor, on-chip time-based measurement, hardware security

## Abstract

This paper presents an in situ time-based sensor designed to provide process authentication. The proposed sensor leverages the inherent metal routing within the chip to measure the RC time-constants of interconnects. However, since the routing metal is typically designed to minimize resistance and capacitance, the resulting small RC time-constants pose a challenge for direct measurement. To overcome this challenge, a “three-configuration” measurement approach is introduced, incorporating two auxiliary components—poly resistor and metal-insulator-metal (MIM) capacitor—to generate three amplified RC time-constants and, subsequently, deduce the routing time-constant. Compared to directly measuring the routing time-constants, this approach reduces measurement error by over 82% while incurring only a 25% area penalty. A straightforward analytical model is presented, taking into account the impact of parasitic capacitances within the discharge path. This analytical model exhibits an excellent concurrence with simulated results, with a maximum difference of only 2.6% observed across all three configurations and a 3.2% variation in the derived routing time-constant. A set of five variants of the time-based sensor are realized using a 130 nm CMOS process. Each variant consists of 44 samples distributed across 11 dies on two wafers. To verify the precision of the proposed sensor, identical resistors and capacitors are fabricated alongside them, forming a direct measurement array (DMA) that is measured using external equipment. After adjusting the routing resistance and capacitance values in the simulations to correspond to the mean values obtained from the DMA measurements, the proposed sensor’s measured results demonstrate a close alignment with simulations, exhibiting a maximum error of only 6.1%.

## 1. Introduction

In recent years, the importance of hardware security has significantly grown, raising concerns about vulnerabilities in semiconductor devices and circuits. These vulnerabilities pose risks such as the potential leakage of sensitive information and the compromise of the entire system’s reliability and performance [1,2,3]. To tackle these vulnerabilities, the concept of the device fingerprint has emerged as a potential solution. In the realm of wireless networks, RF fingerprint, also known as RF distinct native attribute (RF DNA), has been developed to identify and classify authorized devices based on their unique features of the individual devices [4,5,6,7].

Similarly, techniques such as die identification [8,9,10,11] and die authentication [12,13,14,15] have been proposed to enhance the security across the entire life cycle of the global supply chain. Die identification involves assigning a unique serial number to each individual die, while die authentication verifies the device’s origin of manufacturing to ensure its authenticity.

Physical Unclonable Functions (PUFs) offer a promising approach for chip identification by leveraging inherent manufacturing variations in integrated circuits [8,9,10,11,16,17,18,19,20,21,22,23]. PUFs generate unique challenge-response pairs (CRPs) that serve as digital fingerprints, enabling reliable chip identification. Nevertheless, while PUFs excel in die identification, they exhibit shortcomings in die authentication. It is important to note that PUFs’ digital output bits lack correlation with the distinct attributes of the original foundry, thus restricting their potential for die authentication.

To address this issue, a process characterization function (PCF) has been proposed, leveraging the unique process characteristics of the foundry to enable die authentication [12,13,14,15]. For example, the authors in [12] decompose path delays into the delays incurred by logic gates, extracting threshold voltage and effective channel length as distinct signatures for distinguishing between different foundries. Similarly, ref. [13] adapts the successive-approximation-register (SAR) analog-to-digital converter (ADC) architecture, leveraging the mismatch in the metal-oxide-metal (MOM) capacitor as a means of authentication. Another proposed approach, as outlined in [14], involves reducing the activation time (the time it takes for a row of DRAM cells to become accessible) and then observing the resulting error patterns as distinctive authentication characteristics. Likewise, the author in [15] examines the pattern of the partial erase process of Flash memory to identify the fabrication origin. Another approach proposed in [24], a machine learning-based approach is used to infer the fab-of-origin by analyzing production test data (276 probe-test measurements per die) from RF transceivers fabricated in two separate facilities, offering a scalable solution for origin classification.

**Figure 1:**
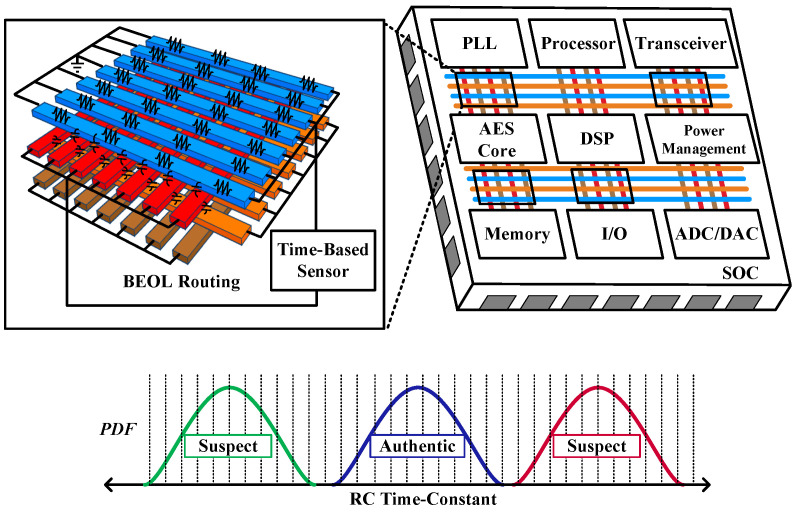
In situ time-based sensors measuring the time-constants of BEOL metal routing for die authentication.

While these papers have proposed various die authentication methods, they pose challenges in seamless integration into other circuits. This is attributed to the necessity for a designated area for PCF circuits, which not only complicates integration but also introduces susceptibility to targeting by attackers. To address this limitation, we propose the use of an in situ time-based sensor that directly measures the RC time-constants of the back-end-of-line (BEOL) metal routing. This sensor can be seamlessly integrated into the functional routing of any system-on-chip (SoC) to provide robust die authentication, as illustrated in Figure 1. To the authors’ best knowledge, this work presents the first in-situ measurement of the RC time-constants of the BEOL metal interconnect.

Additionally, in advanced technology nodes, the RC time-constant of the metal traces increases [25,26], while the time-domain resolution improves, enhancing the sensor authentication accuracy.

The remainder of the paper is organized as follows. Section 2 details the proposed in situ time-based sensor design and its operation. An analytical model of the proposed sensor is presented in Section 3, considering the impact of parasitic capacitances in the discharge path. This model demonstrates a close correlation with simulation results. Section 4 provides the implementation details of the circuit blocks, while Section 5 offers an in-depth analysis of the measurement outcomes. The conclusion is presented in Section 6.

## 2. Proposed Time-Based Sensor

In this section, we introduce the proposed in situ time-based sensor and elucidate its circuit operation. This sensor capitalizes on the chip’s internal metal routing to directly gauge the RC time-constants of interconnects. Since the routing metal is typically optimized to minimize resistance and capacitance, employing this direct measurement strategy results in significant measurement error that diminishes the efficacy of authentication. To address this challenge, a “three-configuration” measurement technique is proposed, and its measurement error is analyzed. Finally, the impact of the process, voltage, and temperature variations is discussed in this section.

### 2.1. Architecture Overview

In the realm of electronic devices, it is common to find resistance-to-digital converters (RDCs) and capacitance-to-digital converters (CDCs) that serve the purpose of estimating input resistance and capacitance. Typically, these devices employ on-chip resistors and capacitors as internal references. Yet, inherent process variations can cause on-chip reference values for resistance and capacitance to shift, requiring the use of an external reference to convert these on-chip references into absolute values [27,28,29]. To overcome this limitation, we propose an in situ time-based sensor that leverages the system clock and its accuracy as a timing reference, eliminating the need for an external reference and allowing the measurement of absolute values of the time-constants without additional calibration.

**Figure 2:**
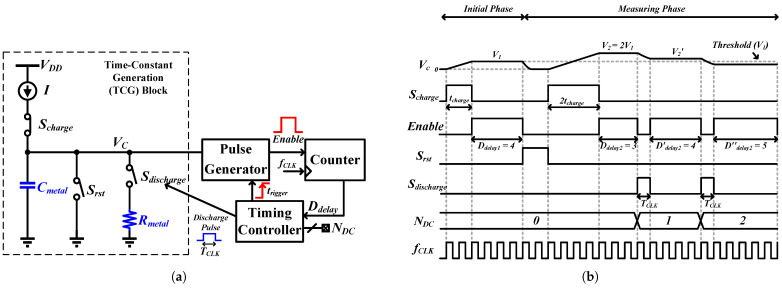
(**a**) Time-based sensor block diagram for direct measurement. (**b**) Timing diagram of the relative signals.

The direct measurement time-based sensor is composed of four main elements, as illustrated in Figure 2a. To begin, a time-constant generation (TCG) block enables an assessment of RC time-constant involving $R_{metal}$ and $C_{metal}$, which represent the resistor and capacitor of the BEOL metal routing. Subsequently, a pulse generator transforms the voltage stored on $C_{metal}$ from the voltage domain into time domain Enable pulses. Following this, a counter acts as a quantizer in the time domain, gauging the duration of these Enable pulses. Lastly, a timing controller coordinates the functions of these elements, ensuring the proper sequence of operations. The RC time-constant can be derived from the accumulated discharge cycle, denoted as $N_{DC}$.

This architecture is primarily digital, with an exception of one analog subcircuit, the current source. Its predominantly digital architecture makes it synthesizable, reducing the integration effort into other circuits and across different technologies.

### 2.2. Circuit Operation

Figure 2a presents the block diagram depicting the direct measurement time-based sensor, while Figure 2b displays a timing diagram illustrating the relative signals. The time-based sensor operates in two distinct phases: an initial phase and a discharging phase. During the initial phase, a current source, *I*, is used to charge the node $V_{c}$ to a threshold voltage, $V_{1}$, by closing the switch $S_{charge}$ for time duration $t_{charge}$. This charging process can be expressed as:

(1) $$\begin{array}{c} V_{1}=\frac{I}{C_{metal}}t_{charge} \end{array}$$

Subsequently, the voltage signal $V_{1}$ is converted into a time-domain signal using a pulse generator, producing a corresponding Enable pulse signal. The pulse width of the Enable signal is then quantized by the system clock, $f_{CLK}$, resulting in a digital code, $D_{delay1}$, that is stored in the timing controller for later processing.

Following the initial phase, the discharging phase begins by closing switch $S_{rst}$ and resetting the $V_{c}$ node to a ground state. Subsequently, the switch $S_{charge}$ is closed for a duration equivalent to twice of the charging period, $2t_{charge}$, leading to the $V_{c}$ node being charged to a voltage $V_{2}$:

(2) $$\begin{array}{c} V_{2}=\frac{I}{C_{metal}}2t_{charge}=2V_{1} \end{array}$$

The $V_{2}$ voltage is transformed into another Enable pulse, which is then quantized to yield $D_{delay2}$. By comparing the digital values $D_{delay1}$ and $D_{delay2}$, it can be determined whether the $V_{c}$ node has fallen below the threshold voltage $V_{1}$. Notably, due to the negative gain of the pulse generator, a higher voltage results in a shorter pulse width, leading to a smaller $D_{delay2}$ when compared to $D_{delay1}$.

As $D_{delay2}$ is found to be smaller than $D_{delay1}$, the timing controller triggers a discharge pulse to turn on the switch $S_{discharge}$ for one clock period, $T_{CLK}$, and increments the count of the discharge cycle, $N_{DC}$, where $N_{DC}$ represents the digital value of the accumulated discharge cycles. While the discharge switch is closed, the charge stored on $C_{metal}$ begins to discharge through the resistor $R_{metal}$. This discharge process follows an exponential decay pattern, resulting in a new voltage, $V_{2}^{\prime }$, on node $V_{c}$. Subsequently, this new voltage generates another Enable pulse along with the corresponding digital code $D_{delay2}^{\prime }$.

The comparison and discharge process continues until the voltage on $V_{c}$ node drops below the threshold voltage $V_{1}$, signaling the conclusion of the discharge phase. The entire discharge process spanning from $V_{2}$ to $V_{1}$ can be represented by:

(3) $$\begin{array}{c} V_{1}\leq V_{2}e^{-N_{DC}T_{CLK}/R_{metal}C_{metal}} \end{array}$$

Here, $N_{DC}T_{CLK}$ represents the total discharge period. Rearranging the terms, the accumulated discharge cycle can be expressed as the corresponding RC time-constant:

(4) $$\begin{array}{c} N_{DC}\approx \frac{R_{metal}C_{metal}\ln\left(2\right)}{T_{CLK}} \end{array}$$

**Figure 3:**
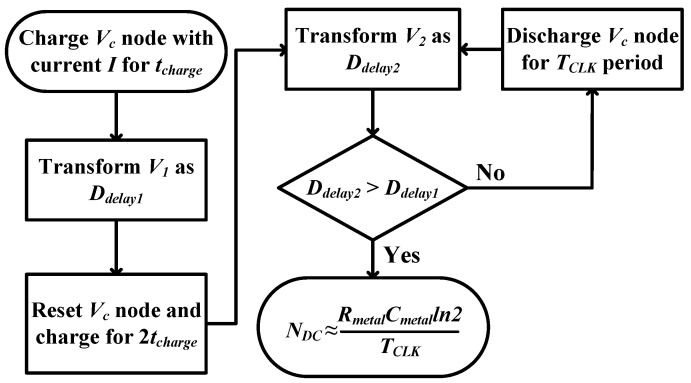
Time-based sensor operation flowchart.

For a detailed visual overview of the described processes, a complete operation flowchart is depicted in Figure 3.

### 2.3. Measurement Error in Direct Measurement

The discrete discharge process described in the preceding section results in quantization error that is pivotal to consider because it can influence the effectiveness of the authentication process. An ideal discharge cycle can be obtained by rewriting Equation (4) and taking the quantization error into consideration:

(5) $$\begin{array}{cc} N_{DC}-q & =\frac{R_{metal}C_{metal}\ln2}{T_{CLK}} \end{array}$$

where *q* (q∈(0,1]) denotes the quantization error. It is important to note that as the discharge process progresses and the $V_{c}$ node voltage drops below the threshold voltage, the quantization error assumes a positive value and is subtracted in the ideal discharge cycle.

The measurement error, expressed as a percentage of the discharge cycle, can then be calculated as:

(6) $$\begin{array}{cc} Error(\%) & =\frac{\left(Meas.-Ideal\right)}{Ideal}\times 100\%\\ & =\frac{\left(N_{DC}\right)-\left(N_{DC}-q\right)}{N_{DC}-q}\times 100\%\\ & =\frac{q}{N_{DC}-q}\times 100\%\\ & \approx \frac{q}{N_{DC}}\times 100\% \end{array}$$

Given that the quantization error ranges from zero to one, maximizing the discharge cycle corresponds to minimizing measurement error. However, the routing time-constant may be in the same order as the clock period, leading to a small discharge cycle and a large measurement error. For example, a long connecting metal trace in a 130 nm CMOS process could have a point-to-point resistance of 6 kΩ ($R_{metal}$) and a total capacitance to ground of 1.5 pF ($C_{metal}$). Assuming a system clock of 500 MHz, this could result in a measurement error of up to 22%. Such a large error significantly diminishes the precision of the measured time-constants, limiting the effectiveness of the RC sensor in authenticating die origins.

### 2.4. Three-Configuration Measurement

In light of the measurement error issue highlighted in the proceeding section, this section presents a “three-configuration” measurement technique aimed at improving the accuracy of the measured RC time-constant. This approach’s primary objective is to reduce the measurement error by utilizing three larger RC time-constants to derive the routing time-constant, $R_{metal}C_{metal}$.

**Figure 4:**
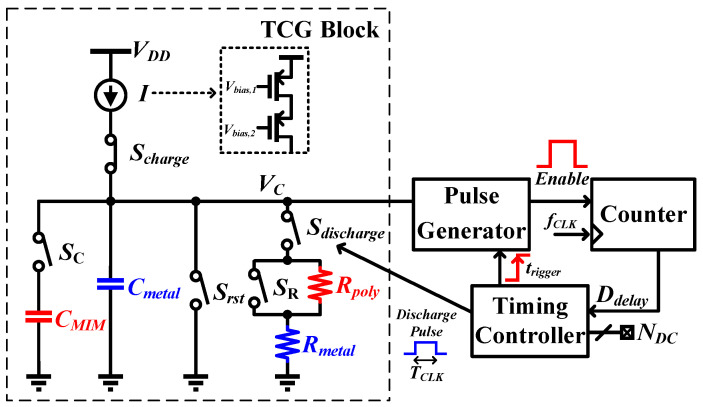
In situ time-based sensor block diagram for three-configuration measurement.

The realization of the “three-configuration” measurement technique involves incorporating two additional components, namely $R_{poly}$ and $C_{MIM}$, as depicted in Figure 4. These elements, realized through a poly-resistor and a metal-insulator-metal (MIM) capacitor, facilitate the achievement of high resistance and capacitance values within a compact area, thereby minimizing area overhead. While it is possible to reduce measurement error by using larger values of $R_{metal}$ and $C_{metal}$, pursuing this approach would result in a significantly greater area consumption compared to the “three-configuration” method.

The “three-configuration” approach follows the same operation concept as described in Section 2.2, albeit employing different components. Three larger time-constants are measured by closing and opening the resistor and capacitor switches, $S_{R}$ and $S_{C}$, in Figure 4, and can be expressed as follows:

(7) $$\begin{array}{c} \left(R_{metal}+R_{poly}\right)C_{metal}\approx \frac{(N_{DC,1})T_{CLK}}{\ln2} \end{array}$$

(8) $$\begin{array}{c} R_{metal}\left(C_{metal}+C_{MIM}\right)\approx \frac{\left(N_{DC,2}\right)T_{CLK}}{\ln2} \end{array}$$

(9) $$\begin{array}{c} \left(R_{metal}+R_{poly}\right)\left(C_{metal}+C_{MIM}\right)\approx \frac{\left(N_{DC,3}\right)T_{CLK}}{\ln2} \end{array}$$

where $N_{DC,n}$ denotes the discharge cycle, with *n* being an index corresponding to one of the three larger RC time-constants. After measuring the three larger time-constants, the desired routing time-constant, $R_{metal}C_{metal}$, can be derived by multiplying the first two larger RC time-constants and dividing by the third:

(10) $$\begin{array}{c} \begin{array}{c} R_{metal}C_{metal}\\ =\frac{\left[\left(R_{metal}+R_{poly}\right)C_{metal}\right]\left[R_{metal}\left(C_{metal}+C_{MIM}\right)\right]}{\left[\left(R_{metal}+R_{poly}\right)\left(C_{metal}+C_{MIM}\right)\right]} \end{array} \end{array}$$

(11) $$\begin{array}{c} R_{metal}C_{metal}\approx (\frac{N_{DC,1}N_{DC,2}}{N_{DC,3}})(\frac{T_{CLK}}{\ln2}) \end{array}$$

where $\frac{N_{DC,1}N_{DC,2}}{N_{DC,3}}$ represents the effective discharge cycle of $R_{metal}C_{metal}$. However, it is important to note that each of the three measurements is subject to its own quantization error. To account for this, Equations (7)–(9) can be adjusted as follows:

(12) $$\begin{array}{c} \left(R_{metal}+R_{poly}\right)C_{metal}=\frac{(N_{DC,1}-q_{1})T_{CLK}}{\ln2} \end{array}$$

(13) $$\begin{array}{c} R_{metal}\left(C_{metal}+C_{MIM}\right)=\frac{\left(N_{DC,2}-q_{2}\right)T_{CLK}}{\ln2} \end{array}$$

(14) $$\begin{array}{c} \left(R_{metal}+R_{poly}\right)\left(C_{metal}+C_{MIM}\right)=\frac{\left(N_{DC,3}-q_{3}\right)T_{CLK}}{\ln2} \end{array}$$

Here, $q_{n}$ (where $0<q_{n}\leq 1$) represents the quantization error associated with each of the three large time-constants. By substituting Equations (12)–(14) into Equation (10), the routing time-constant $R_{metal}C_{metal}$, alongside its corresponding quantization error, can be determined. The complete derivation can be found in Appendix A.

(15) $$\begin{array}{c} \begin{array}{c} R_{metal}C_{metal}\\ =\frac{\left[\left(R_{metal}+R_{poly}\right)C_{metal}\right]\left[R_{metal}\left(C_{metal}+C_{MIM}\right)\right]}{\left[\left(R_{metal}+R_{poly}\right)\left(C_{metal}+C_{MIM}\right)\right]}\\ \approx (\frac{N_{DC,1}N_{DC,2}}{N_{DC,3}}-\frac{q_{1}N_{DC,2}+q_{2}N_{DC,1}}{N_{DC,3}})(\frac{T_{CLK}}{\ln2}) \end{array} \end{array}$$

It is worth noting that the second term, $\frac{q_{1}N_{DC,2}+q_{2}N_{DC,1}}{N_{DC,3}}$, in the above equation accounts for the quantization error arising from the “three-configuration” measurement technique. To significantly mitigate this quantization error, the values of the auxiliary components in this prototype are selected to be 10× those of the routing resistance and capacitance.

(16) $$\begin{array}{c} R_{poly}=10R_{metal}\;\;\;\mathrm{and}\;\;\;C_{MIM}=10C_{metal} \end{array}$$

This selection of auxiliary components results in $\left(R_{poly}+R_{metal}\right)C_{metal}=R_{metal}(C_{MIM}$$+\;C_{metal})=11R_{metal}C_{metal}$. Consequently, the corresponding discharge cycle becomes 11 times larger than that of a direct measurement ($N_{DC,2}=N_{DC,1}=11N_{DC}$). This choice of selection in the auxiliary components directly impacts the measurement error of the derived $R_{metal}C_{metal}$, which simplifies to

(17) $$\begin{array}{cc} Error(\%) & =\frac{q_{1}N_{DC,2}+q_{2}N_{DC,1}}{N_{DC,1}N_{DC,2}-q_{1}N_{DC,2}-q_{2}N_{DC,1}}\times 100\%\\ & \approx \frac{2q_{1}}{11N_{DC}}\times 100\% \end{array}$$

Compared to the measurement error obtained from the direct measurement in Equation (6), the three-configuration measurement method reduces the measurement error by 82% through the use of auxiliary components with 10× resistance and capacitance values. Despite using additional elements, it only results in a 25% increase in the overall footprint, owing to the high resistance and capacitance density achieved by the poly resistor and the MIM capacitor in the target 130 nm CMOS process.

### 2.5. Process, Voltage, and Temperature Variations

The time-based sensor employs a current source to generate two proportional voltages and measures the time-constants by monitoring the discharge period. As long as the ratio of the charging periods is precise, the proportional voltages are accurate, leading to precise time-constant values. Despite the absolute value of the current source varies due to process, voltage, and temperature (PVT) variations, the approach remains robust as it primarily relies on the accuracy of the on-chip clock rather than requiring a precise on-chip voltage or current reference. Furthermore, given the sensor’s brief operational duration, we can safely assume that voltage and temperature remain stable without experiencing transient variations across the circuit’s operation.

## 3. Analytical Model

In this section, an analytical model of the TCG block is introduced. The model is both comprehensive and straightforward, effectively providing insights into how the parasitic capacitances associated with MOSFET switches and resistors influence the sensor’s discharge cycle. The accuracy of this model is validated through a comparison with the circuit simulation results.

### 3.1. Charge Redistribution Effect

One critical factor that significantly impacts the discharge cycle of the time-based sensor is the presence of parasitic capacitances within the discharge path, specifically associated with the resistors ($R_{metal}$ and $R_{poly}$) and the MOSFET switches ($S_{discharge}$ and $S_{R}$). To establish a straightforward yet effective model, the parasitics of the $R_{metal}$ and $R_{poly}$, initially following a distributed RC model, are simplified to a π-model with two lumped capacitors, $C_{sub}$, at both ends.

Similarly, the junction capacitances, $C_{j}$, of the MOSFET switches are modeled as capacitors at the source and drain terminals. To streamline the model, the overlapped capacitance, $C_{ol}$, and channel capacitance, $C_{channel}$, are simplified to two capacitors, $C_{gs}$ and $C_{gd}$, representing the gate-to-source and gate-to-drain capacitances, respectively.

**Figure 5:**
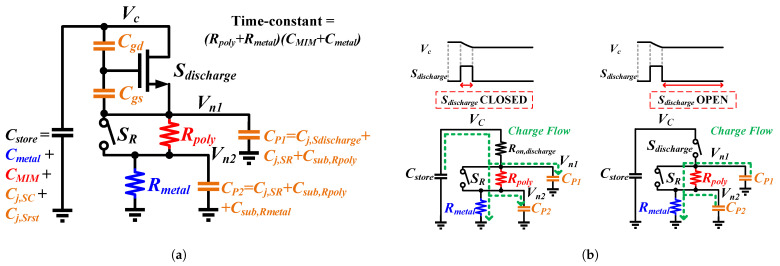
(**a**) TCG block with two lump capacitors simplifying the parasitic capacitances of resistors and MOSFET switches. (**b**) Charge flow of the TCG block.

Considering that $C_{j}$ and $C_{sub}$ are both connected to the substrate, they can be merged. Consequently, the parasitic capacitances at internal nodes, $V_{n1}$ and $V_{n2}$, within the discharge path can be simplified into two lump parasitic capacitors, $C_{p1}$ and $C_{p2}$, as illustrated in Figure 5a. Specifically, $C_{p1}$ includes the parasitic capacitance from $S_{discharge}$, $S_{R}$, and $R_{poly}$, while $C_{p2}$ contains the parasitics of $S_{R}$, $R_{poly}$, and $R_{metal}$.

Additionally, to streamline the symbols, $C_{metal}$, $C_{MIM}$, and the parasitic capacitance of $S_{C}$ and $S_{rst}$ are combined into a single capacitor, denoted as $C_{store}$, which is responsible for storing the charge on the $V_{c}$ node, as shown in Figure 5a. The ensuing discussion will focus on the effects of $C_{p1}$ and $C_{p2}$, while the impacts of $C_{gs}$ and $C_{gd}$ of the discharge switch, which results in a clock feedthrough effect, will be covered subsequently.

With a comprehensive understanding of the key parasitic capacitances in the TCG block, we now delve into the details of the discharge process. Upon closure of the switch $S_{discharge}$, the charge stored on $C_{store}$ redistributes, flowing through $R_{poly}$ and $R_{metal}$ to ground as well as to the parasitic capacitors, $C_{p1}$ and $C_{p2}$, as illustrated in Figure 5b. Voltages $V_{n1}$ and $V_{n2}$ are determined by the resistor-divider ($R_{on,discharge}$, $R_{poly}$, and $R_{metal}$) present in the discharge path. In contrast, when the discharge switch is opened, the charge flow from $C_{store}$ ceases, and the charge stored on $C_{p1}$ and $C_{p2}$ discharges to ground, as depicted in Figure 5b. Given that the discharge switch OFF time being significantly longer than the time-constants of the parasitic capacitors, $V_{n1}$ and $V_{n2}$ nodes are reset to ground when the discharge switch is opened. Therefore, each cycle of closing and opening the discharge switch results in an additional loss of charge from $C_{store}$, calculated as $C_{p1}V_{n1}+C_{p2}V_{n2}$. As the cumulative number of discharge cycles increases, the charge redistribution effect becomes more pronounced, leading to an overall reduction in the number of discharge cycles.

An analytical model of the TCG block can be developed by establishing Kirchhoff’s Current Law (KCL) for nodes $V_{c}$, $V_{n1}$, and $V_{n2}$:

(18) $$\begin{array}{c} \left\{\begin{array}{c} -\left(C_{metal}+C_{MIM}\right)\frac{dV_{c}\left(t\right)}{dt}=\frac{V_{c}\left(t\right)-V_{n1}\left(t\right)}{R_{on,discharge}}\\ \frac{V_{c}\left(t\right)-V_{n1}\left(t\right)}{R_{on,discharge}}=\frac{V_{n1}\left(t\right)-V_{n2}\left(t\right)}{R_{poly}}+C_{p1}\frac{dV_{n1}\left(t\right)}{dt}\\ \frac{V_{n1}\left(t\right)-V_{n2}\left(t\right)}{R_{poly}}=\frac{V_{n2}\left(t\right)}{R_{metal}}+C_{p2}\frac{dV_{n2}\left(t\right)}{dt} \end{array}\right. \end{array}$$

where $R_{on,discharge}$ is the on resistance of the discharge switch. To accurately capture the discharge behavior, the analytical model is solved for every discharge cycle.

### 3.2. Clock Feedthrough Effect

In addition to the charge redistribution effect caused by parasitic capacitances in the discharge path, the two parasitic capacitors $C_{gs}$ and $C_{gd}$ of the discharge switch induce a clock feedthrough effect. This impact alters the voltages at nodes $V_{c}$ and $V_{n1}$ during both the low-to-high and high-to-low transitions of the discharge pulse. While $C_{gs}$ and $C_{gd}$ are voltage-dependent, the time-based sensing method inherently averages this nonlinearity during operation, and the analytical model uses these average values to maintain compactness while preserving accuracy.

**Figure 6:**
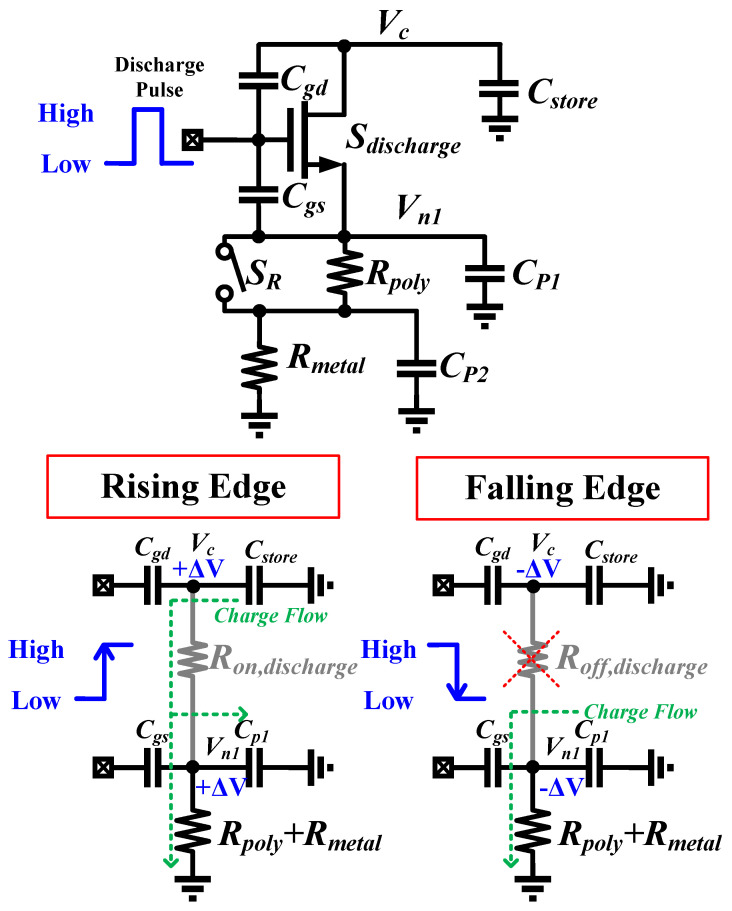
Clock feedthrough effect on the discharge switch, $S_{discharge}$.

Upon the rising edge of the discharge pulse arrival, the voltages at nodes $V_{c}$ and $V_{n1}$ increase due to capacitive coupling. This increase in voltage slows down the discharge process on $C_{store}$, as depicted in Figure 6. Conversely, during the falling edge, the voltages at nodes $V_{c}$ and $V_{n1}$ decrease, resulting in an accelerated discharge process.

It should be emphasized that while the low-to-high and high-to-low transitions sum a zero effect on $V_{c}$ node, this is not the case for the $V_{n1}$ node. When the falling edge of the discharge pulse arrives and $S_{discharge}$ opens, the discharging path of $C_{store}$ is disconnected. Hence, the decrease in voltage on the $V_{n1}$ node does not affect $C_{store}$ and merely accelerates the reset process. To incorporate this clock feedthrough effect into the analytical model, a non-zero initial condition is used for the $V_{n1}$ node when solving the equations. This approach captures the impact of the clock feedthrough effect while preserving the simplicity of the analytical model.

### 3.3. Simulation Results

**Figure 7:**
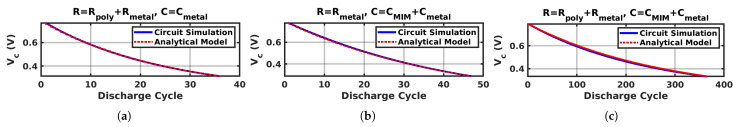
Simulation results of the analytical model and circuit simulations. (**a**) $R=R_{poly}+R_{metal}$, $C=C_{metal}$ (**b**) $R=R_{metal}$, $C=C_{MIM}+C_{metal}$ (**c**) $R=R_{poly}+R_{metal}$, $C=C_{MIM}+C_{metal}$.

In order to validate the effectiveness of the proposed analytical model, a comparison is drawn between its simulation results and those obtained from circuit simulations. The simulations were performed using a 130 nm CMOS process with extracted parasitics included. As illustrated in Figure 7, a close alignment can be observed between the results derived from the analytical model and the circuit simulations across all three larger RC time-constants. A summary of the simulation results for these RC time-constants is provided in Table 1. Notably, the difference between the analytical model and the circuit simulations is maintained below 3.2%, underscoring the high level of accuracy attained by the model.

## 4. Implementation Details

This section delves into the design and implementation of the in situ time-based sensors. In order to address the impact of process variation, Monte Carlo simulations, including extracted parasitic, are employed to estimate the post-fabrication results. A thorough examination and interpretation of the simulation outcomes are also presented.

### 4.1. TCG Block

The architecture of the TCG block is illustrated in Figure 4. For simplicity, the bias circuitry is omitted from the figure. To minimize parasitic capacitances, all switches are implemented with minimum length NMOS devices. It is important to note that while regular NMOS devices are used for the $S_{R}$ switch, thick gate devices are chosen for the remaining switches connected to the $V_{c}$ node to minimize leakage current. Additionally, both the $S_{discharge}$ and $S_{R}$ switches are designed with turn-on resistance significantly smaller than the $R_{metal}$, ensuring that the time-constant measurement is dominated by the $R_{metal}$, $R_{poly}$, $C_{metal}$, and $C_{MIM}$.

### 4.2. Pulse Generator

**Figure 8:**
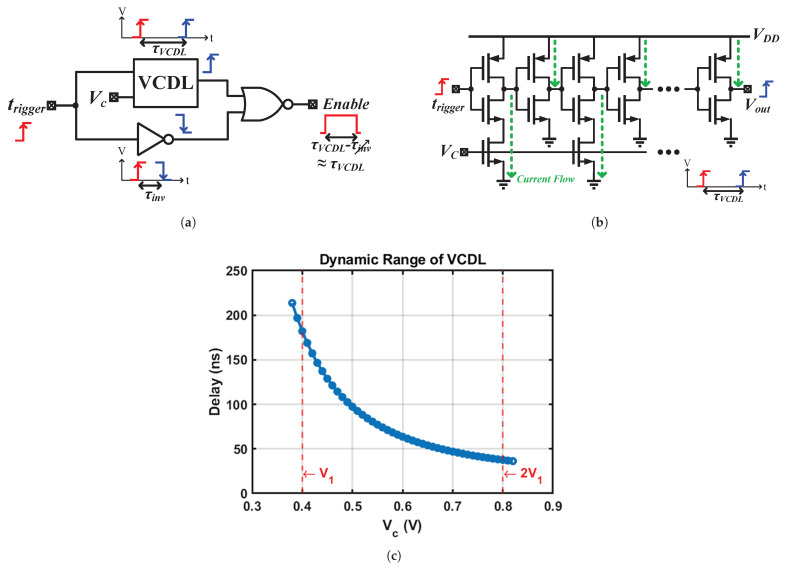
(**a**) Pulse generator. (**b**) Voltage-controlled delay line (VCDL). (**c**) Dynamic range of VCDL.

The objective of the pulse generator (Figure 4) is to convert the voltage $V_{c}$ from voltage domain to an equivalent time domain Enable pulse, which is subsequently qunatized using the system clock. This time-domain measurement approach contrasts with direct voltage measurement using an ADC, which would require an accurate on-chip voltage reference and incurs area overhead, making the time-domain method a more efficient alternative. This conversion is achieved through the combination of a voltage-controlled delay line (VCDL), an inverter, and a NOR gate, as illustrated in Figure 8a. When $V_{c}$ is settled, a trigger signal, $t_{trigger}$, is dispatched from timing controller to the pulse generator. The VCDL and the inverter transmit the transition to their respective outputs with distinct propagation delays, as shown as $\tau_{VCDL}$ and $\tau_{inv}$, respectively. Subsequently, the delay difference is converted into an Enable pulse by the NOR gate, which then proceeds to a counter for quantization. Since $\tau_{inv}$ is negligible compared to $\tau_{VCDL}$, the Enable pulse width is approximately equal to $\tau_{VCDL}$.

Figure 8b shows the schematic of the VCDL, consisting of 26 current-starved inverters followed by 26 regular inverters in an alternating pattern. By limiting the discharge current via the $V_{c}$ voltage, the propagation delay of the VCDL can be effectively controlled. The dynamic range of VCDL, covering the voltage range from $V_{1}$ to $2V_{1}$, is depicted in Figure 8c and spans from 37 ns to 182 ns.

### 4.3. Counter

**Figure 9:**
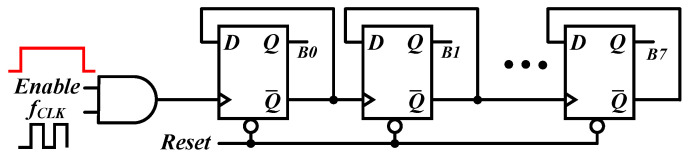
Counter.

To digitize the Enable pulse width, a sequence of D-flip-flops (DFFs) is connected in series, with their outputs (QB) looped back to the data (D) to form a counter. An AND gate is employed at the counter’s front end as an enabling mechanism, as depicted in Figure 9. Considering the system clock is 500 MHz, an eight-bit counter is chosen to cover the dynamic range of the VCDL, where B0 and B7 are the least-significant bit (LSB) and the most-significant-bit (MSB) of the $D_{Delay}$, respectively. This counter design provides a simple yet effective approach to digitize the Enable pulse width.

### 4.4. Timing Controller

**Figure 10:**
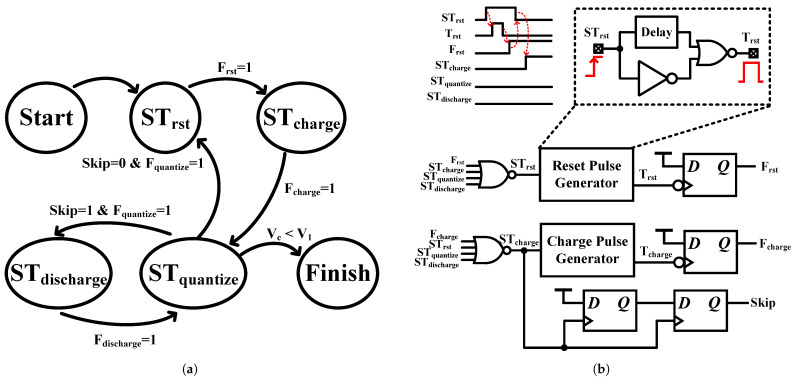
(**a**) State machine of the timing controller. (**b**) Partial timing controller circuits for simplicity.

The timing controller coordinates the sequential operations of each block within the system. Illustrated in Figure 10a, the state machine of the timing controller comprises four distinct states: $ST_{rst}$ (resetting the $V_{c}$ node), $ST_{charge}$ (charging the $V_{c}$ node), $ST_{quantize}$ (quantizing the voltage on the $V_{c}$ node and comparing it to the threshold voltage), and $ST_{discharge}$ (discharging the $V_{c}$ node). Each state is associated with a finished signal, denoted as $F_{n}$, where *n* represents one of the four states. Additionally, there is a Skip signal that determines whether the system is in the discharging phase. After the state machine enters the $ST_{charge}$ state twice, the Skip signal is raised, restricting the state machine’s transitions to alternate between the $ST_{quantize}$ and $ST_{discharge}$ states.

Upon initialization of the time-based sensor, $ST_{rst}$ is set high, generating a reset pulse, $T_{rst}$, which resets the voltage on the $V_{c}$ node, as demonstrated in Figure 10b. Subsequently, the falling edge of the pulse triggers a DFF, raising the finished signal $F_{rst}$ to a high state. Once activated, this signal lowers $ST_{rst}$ and initiates the $ST_{charge}$ state. For clarity, only two states logic are shown in Figure 10b, yet the remaining states are implemented in a similar manner.

### 4.5. Time-Based Sensor

For the sake of design validation, a set of five RC variants with different resistor and capacitor values have been implemented. The five variants, denoted as *R-hC*, *hR-C*, *R-C*, *2R-C*, and *R-2C*, represent combinations of resistors and capacitors at 0.5× (notated as *h*), 1×, and 2× their nominal values. In these configurations, the nominal values for $R_{metal}$ and $C_{metal}$ are 6 kΩ and 1.5 pF, respectively. As discussed in Section 2.4, the auxiliary components are chosen to be 10 times larger than $R_{metal}$ and $C_{metal}$ to significantly reduce the quantization error, leading to $R_{poly}$ at 60 kΩ and $C_{MIM}$ at 16 pF for the *R-C* variant. Detailed parameters for *R-C* variant are listed in Table 2.

To estimate the distribution of the discharge cycle after fabrication, the parasitic extracted time-based sensors are simulated with 200-point Monte Carlo simulations. The simulation results for the three configurations and the derived routing time constant are summarized in Table 3. For the purpose of brevity, only the *R-C* variant is reported. The simulated mean value (μ) and standard deviation (σ) for each configuration are tabulated, providing a statistical characterization of the discharge cycle distribution. The $RC_{design}$ column represents the RC time-constants used in simulations, while the $RC_{cal}$ column provides the calculated time-constants derived from the mean value of the discharge cycle. Discrepancies between these values are presented as a percentage difference in the Error column.

As discussed in Section 3.1, the charge redistribution effect is influenced by several factors, including the storing capacitor $C_{store}$, the parasitic capacitors $C_{p1}$ and $C_{p2}$, the resistors $R_{poly}$ and $R_{metal}$, and the number of the discharge cycle. Consequently, while the calculated time-constant in each configuration is smaller than the designed value, the level of the charge redistribution effect varies among them. For instance, the $R_{metal}\left(C_{metal}+C_{MIM}\right)$ configuration, with an error of 11.5%, exhibits less discrepancy than the $\left(R_{metal}+R_{poly}\right)C_{metal}$ configuration, which has an error of 26.6%, due to its larger storing capacitor. However, in the $\left(R_{metal}+R_{poly}\right)\left(C_{metal}+C_{MIM}\right)$ configuration, despite having a large storing capacitor, it accumulates the highest number of discharge cycles. This frequent occurrence of charge redistribution results in more significant charge loss from $C_{store}$ and, consequently, leads to the largest error among the three configurations.

The discharge cycle of the routing time-constant, $R_{metal}C_{metal}$, is derived in Table 3 based on Equation (11). As expected from Equation (17), the error is significantly reduced through the calculation of the quotient $\left(R_{metal}+R_{poly}\right)C_{metal}\times R_{metal}\left(C_{metal}+C_{MIM}\right)/\left(R_{metal}+R_{poly}\right)\left(C_{metal}+C_{MIM}\right)$, thereby mitigating the influence of the charge redistribution effect. Consequently, the derived routing time-constant yields a more accurate result with a minimal error of 0.89%.

### 4.6. Direct Measurement Array

**Figure 11:**
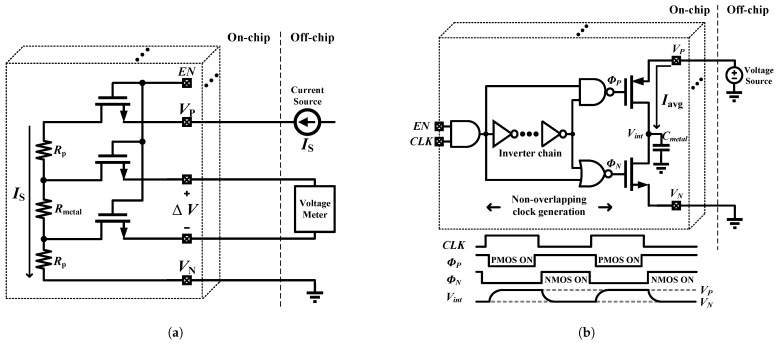
Direct measurement array structure (**a**) Resistor array. (**b**) Capacitor array.

In addition to the in situ time-based sensors, standalone layouts of $R_{metal}$ and $C_{metal}$ structures have been fabricated alongside the sensors, resulting in a direct measurement array (DMA). The structures of DMA resistor array and DMA capacitor array are depicted in Figure 11, and detailed in [30]. Notably, since external equipment is used to measure the DMA, the precise outcomes from the DMA are utilized as a benchmark for the validation of the accuracy of the time-based sensors.

The structure of the DMA resistor array is illustrated in Figure 11a. A single cell is enabled at a time by activating its corresponding EN signal. An accurate external current source is then utilized to measure the resistance values of $R_{metal}$. Notably, owing to the high input impedance of the voltage meter, the current $I_{s}$ exclusively flows through $R_{metal}$ and the parasitic resistance, $R_{p}$, without being drawn into the voltage meter. Concurrently, the voltage meter measures the voltage across the $R_{metal}$, and the resistance vale of $R_{metal}$ can be determined by: $R_{metal}=\Delta V/I_{s}$. It is noteworthy that the measurement accuracy may be influenced by the leakage of the MOSFET switches. To mitigate this issue, thick gate devices are adopted in this structure. Simulations indicate that the leakage current of thick gate device is at a pA level.

The structure of the DMA capacitor array is depicted in Figure 11b. Similar to the resistor DMA, a single cell is activated at a time through the utilization of an EN signal. The operation involves the input of a clock signal, CLK, into a non-overlapping clock circuit, which generates two distinct clock phases, $\phi_{P}$ and $\phi_{N}$. These clock signals ensure the PMOS and NMOS do not turn on simultaneously. Specifically, when $\phi_{P}$ is in its low state, the PMOS is in an ON-state, while the NMOS is in an OFF-state, thereby allowing a voltage source, $V_{P}$, to charge the $C_{metal}$ capacitor. Conversely, when $\phi_{N}$ is in its high state, the PMOS turns off, and the NMOS turns on, leading to the discharge of $C_{metal}$ to a ground state, $V_{N}$. The value of $C_{metal}$ can then be determined by measuring the average current, $I_{avg}$, drawn from the voltage source $V_{P}$, utilizing the formula $C_{metal}=I_{avg}\times T_{CLK}/\left(V_{P}-V_{N}\right)$ [31]. Note that even though the clock, CLK, has a 50% duty cycle, the on-time of the PMOS and NMOS are not equal to half of the clock period due to the propagation delay of the inverter chain. This discrepancy can introduce a measured error in the determination of $C_{metal}$. To mitigate this error, half of the clock period needs to be significantly larger than the delay of the inverter chain. Simulations show that the delay of the inverter chain is about 130 ps.

## 5. Measurement Results

**Figure 12:**
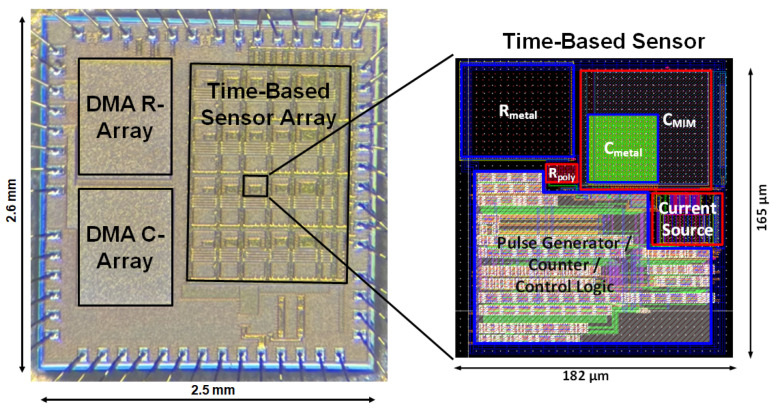
Die photo highlighting the time-based sensor layout.

An array of the five time-based sensor variants has been fabricated in a 130 nm CMOS technology with a 1.5 V supply voltage. The die photo, shown in Figure 12, reveals a total area of 2.5 mm × 2.6 mm, accommodating 81 DMA-resistance cells, 108 DMA-capacitance cells, and 20 time-based sensors.

The layout of a single time-based sensor (*R-C* variant) occupies an area of 182 µm × 165 µm.

Measurement data have been collected from 11 chips, with four from one wafer and the remaining seven from another wafer.

To ensure accuracy, each reported data point is the average of three measurements which helps suppress the impact of noise.

### 5.1. Direct Measurement Array—Resistor Array

The DMA-resistor array comprises three types of resistor cells labeled as *hR*, *R*, and *2R*, with values of 3 kΩ, 6 kΩ, and 12 kΩ, respectively. These values correspond to 0.5×, 1×, and 2× of the nominal resistance values. Each cell type includes 27 identical elements, bringing the total to 81 elements within the resistor array.

The external current source is supplied by a Keithley-2400 unit (Keithley Instruments, Inc., Solon, OH, USA), configured to provide a current of 10 µA, while the voltage measurements are carried out using a Keysight-34420A device (Keysight Technologies, Inc., Santa Rosa, CA, USA). Given that the leakage current observed from simulations is at the pA level, the impact of the leakage current is negligible.

**Figure 13:**
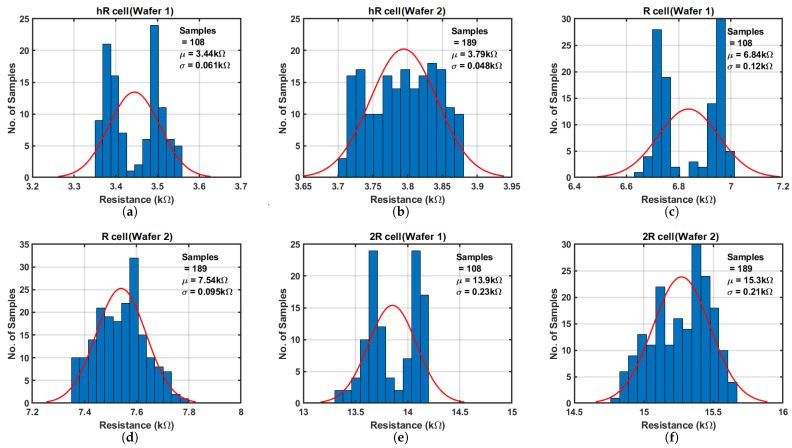
Measurement results of DMA-resistor array. (**a**,**c**,**e**) results are from the first wafer, and (**b**,**d**,**f**) from the second wafer.

Measured results from the first wafer, encompassing four chips, are presented in Figure 13a,c,e. The displayed bimodal shape of the histogram is attributed to die-to-die variation and the small sample size. As the sample size increases, it is expected that the distribution would converge toward a Gaussian distribution. Similarly, results from the second wafer, involving seven chips, are plotted in Figure 13b,d,f. Notably, the second wafer’s three resistance cell mean values consistently exhibit a 10% elevation compared to those of the first wafer, likely due to process variation. When compared to circuit simulations in the typical-typical (TT) corner, the mean values of the measured results across two wafers are approximately 22% higher for all three resistor cells, as summarized in Table 4. This discrepancy may be attributed to the difference between models and real manufacturing process as well as process variations. This discrepancy highlights the critical need for the DMA measurements, as simulation results alone are insufficient.

### 5.2. Direct Measurement Array—Capacitor Array

The DMA-capacitor array comprises three types of capacitor cells denoted as *hC*, *C*, and *2C*, with values of 0.73 pF, 1.45 pF, and 2.88 pF, respectively, corresponding to 0.5×, 1×, and 2× capacitance values. Each cell type contains 36 identical elements, yielding a total 108 elements within the capacitor array.

The clock signal is generated using Agilent E8267D equipment (Keysight Technologies, Inc., Santa Rosa, CA, USA), employing on-chip buffers to produce a square wave with a 50% duty cycle at 20 MHz.

**Figure 14:**
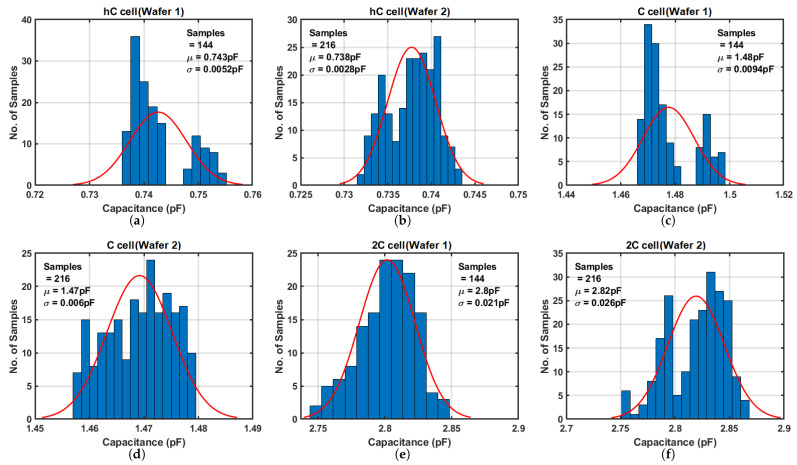
Measurement results of DMA-capacitor array. (**a**,**c**,**e**) results are from the first wafer, and (**b**,**d**,**f**) from the second wafer.

The voltage sources $V_{P}$ and $V_{N}$ are provided by Keithley-2400 unit set at 1.5 V and 0 V, respectively. Since half of the clock period is 25 ns, significantly larger than the delay of the inverter chain, the measured error due to the shortened charging period is negligible. Figure 14 presents the measured results separately for each wafer. Due to die-to-die variation and a small sample size, the histograms deviate from a Gaussian distribution. Notably, unlike the resistor array, the mean values across the three capacitor cells from both wafers are notably similar. When compared to circuit simulations in the TT corner, the mean values of the measured results closely match, with a maximum error of 2.5%. These results are summarized in Table 5.

### 5.3. Time-Based Sensors

In addition to the DMA resistor and capacitor arrays, an array of time-based sensors has been fabricated. This array consists of five RC variants, each comprising four sensor units, resulting in a total of 20 sensors on a single chip.

The *R-C* variant operates at 500 MHz and consumes 16.2 mW from a 1.5 V supply. It is worth noted that the time-based sensors only need to be activated when an authentication verification is required, hence the power consumption is not a significant concern.

Based on DMA results, the measured mean values of $R_{metal}$ and $C_{metal}$ deviated from the circuit simulation values, especially for the resistor values. To account for this deviation, adjustments are made to the $R_{metal}$ and $C_{metal}$ values in the time-based sensors’ simulations using the mean values measured from the DMA. This modification led to closely matched results between simulations and measurements across the five variants, with a maximum error of 6.1%, as summarized in Table 6.

Discrepancies between the simulations and the measurements can be attributed to within-die variation (DMA vs. time-based sensor) and variations in the parasitic capacitance values within the discharge path, which are not accounted for in the simulation results. These fluctuations can lead to different levels of charge redistribution, resulting in varying discharge cycles. It is important to emphasize that the observed maximum deviation of 6.1% does not solely reflect sensor measurement error but instead encompasses the cumulative impact of effects such as intra-die process variations and model limitations.

For context, prior work by Jin et al. [32] has shown that RC time constants in a 55 nm CMOS process can vary by up to ±50% due to PVT effects. Compared to this baseline, the 6.1% deviation observed here reflects significantly higher measurement fidelity than the intrinsic variation within a single foundry. It therefore stands to reason that the proposed method is capable of distinguishing devices fabricated in different foundries, where variation is expected to be even greater.

**Figure 15:**
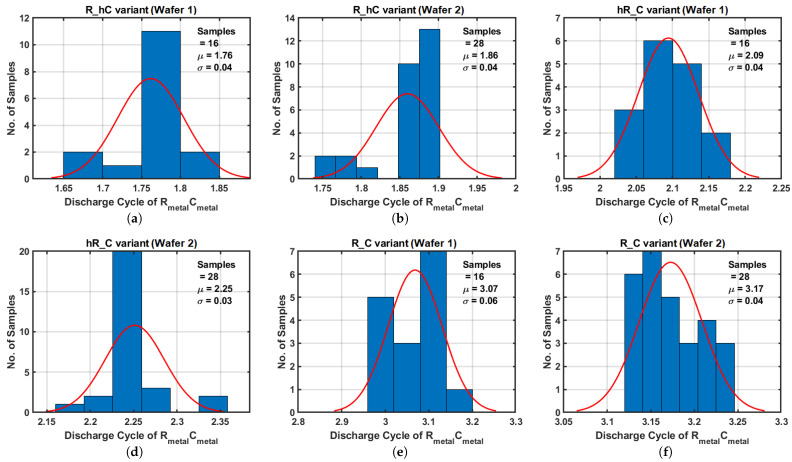

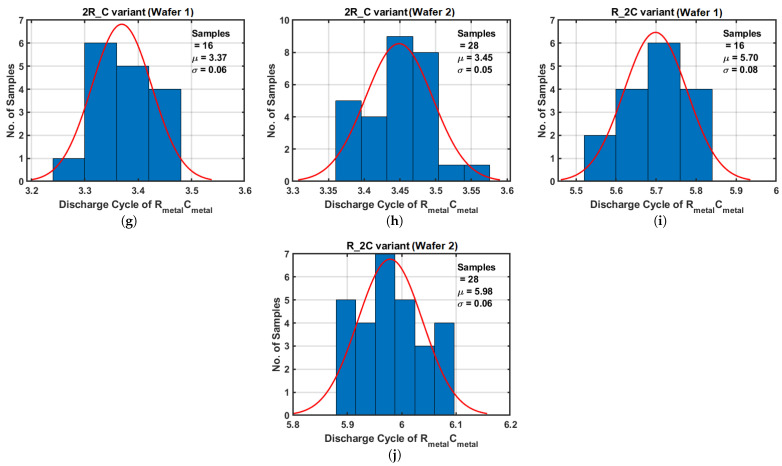
Measurement results of time-based sensors. (**a**,**c**,**e,g**,**i**) results are from the first wafer, and (**b**,**d**,**f**,**h**,**j**) from the second wafer.

The distribution of the derived discharge cycles for $R_{metal}C_{metal}$ is shown in Figure 15, with separate data for each wafer. Notably, the discharge cycles of the second wafer consistently exceed those of the first wafer, aligning with the DMA resistor array results. Similar to the histograms shown for the DMA, as the sample size increases, the distribution is expected to converge toward a Gaussian distribution.

## 6. Conclusions

This paper introduces an in situ time-based sensor capable of characterizing the RC time constants of the BEOL routing, enabling process attestation. Leveraging inherent process variations during manufacturing, the sensor facilitates die authentication. To address the resolution issue arising from a slow clock frequency, a three-configuration measurement method is proposed. Theoretical analysis shows that utilizing two auxiliary components with 10× resistance and capacitance values can reduce measurement error by 82%.

An analytical model is developed to study the impacts of each component in the TCG block on the discharge cycle, including the parasitic capacitances of the MOSFET switches and those of the resistors in the discharge path. The model simplifies these parasitic capacitances while incorporating charge redistribution and clock feedthrough effects, achieving close alignment with circuit simulations.

To validate the accuracy of the time-based sensors, a DMA with identical layouts of $R_{metal}$ and $C_{metal}$ alongside the sensors is fabricated. The DMA enables accurate resistance and capacitance measurement using external equipment. By updating the $R_{metal}$ and $C_{metal}$ values in the time-based sensor simulations in accordance with the DMA results, a strong correlation between the simulations and measurements is observed, validating the effectiveness of the proposed sensor.

In summary, the time-based sensor presented in this work offers a promising solution for process attestation, contributing to the advancement of secure and reliable hardware systems.

## Figures and Tables

**Table 1:** Discharge cycles of the analytical model and Cadence simulation.

Configuration		Analytical Model $N_{\mathrm{DC}}$	Circuit Simulation $N_{\mathrm{DC}}$ ^a^	Model Error
$R_{poly}+R_{metal}$	$C_{metal}$	26	26	<1%
$R_{metal}$	$C_{MIM}+C_{metal}$	32	32	<1%
$R_{poly}+R_{metal}$	$C_{MIM}+C_{metal}$	277	270	<2.6%
$R_{metal}$	$C_{metal}$	3.0 ^b^	3.1 ^b^	<3.2%

^a^ Simulated with parasitics extracted. ^b^ Derived discharge cycle from Equation (11).

**Table 2:** Design parameters of *R-C* variant.

Parameter	Value	Parameter	Value
$V_{DD}$	1.5 V	$C_{MIM}$	16 pF
$f_{CLK}$	500 MHz	$C_{metal}$	1.5 pF
$R_{poly}$	60 kΩ	$V_{1}$	0.4 V
$R_{metal}$	6 kΩ	$V_{2}$	0.8 V

**Table 3:** RC time-constant measurement results.

Configuration		${\mathrm{RC}}_{\mathrm{design}}$	$N_{\mathrm{DC}}\left(\mu \right)$	Std (σ)	${\mathrm{RC}}_{\mathrm{cal}}$ ^*a*^	Error ^*b*^
$R_{metal}+R_{poly}$	$C_{metal}$	99 ns	25.9	3.6	74.7 ns	26.6%
$R_{metal}$	$C_{metal}+C_{MIM}$	105 ns	32.2	4.4	92.9 ns	11.5%
$R_{metal}+R_{poly}$	$C_{metal}+C_{MIM}$	1155 ns	269.8	16.7	778.5 ns	32.6%
$R_{metal}$	$C_{metal}$	9 ns	3.09 ^*c*^	0.64	8.92 ns	0.89%

^*a*^ Measured RC time-constant, $RC_{cal}=N_{DC}*T_{CLK}/\ln\left(2\right)$. ^*b*^ $Error=\left(RC_{design}-RC_{cal}\right)/RC_{design}$. ^*c*^ Derived discharge cycle from Equation (11).

**Table 4:** Summary of DMA-Resistance array.

Cell	Simulation Results	Measurement Results	Process Shift
*hR*	3 kΩ	3.67 kΩ	22%
*R*	6 kΩ	7.28 kΩ	21%
*2R*	12 kΩ	14.8 kΩ	23%

**Table 5:** Summary of DMA-Capacitance array.

Cell	Simulation Results	Measurement Results	Process Shift
*hC*	0.73 pF	0.74 pF	1.4%
*C*	1.45 pF	1.47 pF	1.4%
*2C*	2.88 pF	2.81 pF	−2.5%

**Table 6:** Summary of time-based sensors.

Configuration		Simulation		Measurement		Error ^b^
**R Cell**	**C Cell**	$N_{\mathrm{DC}}$ ^a^ (μ)	**Std** (σ)	$N_{\mathrm{DC}}$ ^a^ (μ)	**Std** (σ)	
*R*	*hC*	1.91	0.06	1.82	0.063	4.9%
*hR*	*C*	2.24	0.06	2.19	0.085	2.2%
*R*	*C*	3.32	0.07	3.13	0.069	6.1%
*2R*	*C*	3.45	0.06	3.42	0.063	0.8%
*R*	*2C*	5.88	0.10	5.88	0.15	0%

^a^ Derived discharge cycle of $R_{metal}C_{metal}$ from Equation (11). ^b^ Error between mean values of the simulation and measurement.

## Data Availability

Data are contained within the article.

## Appendix A. Three-Configuration Measurement Derivation

In three-configuration measurement, the routing time-constant, $R_{metal}C_{metal}$, is derived by measuring three larger RC time-constants. By substituting Equations (12)–(14) into Equation (10), the routing time-constant, $R_{metal}C_{metal}$, can be expressed as:

(A1) $$\begin{array}{c} \begin{array}{c} R_{metal}C_{metal}\\ =\frac{\left[\left(R_{metal}+R_{poly}\right)C_{metal}\right]\left[R_{metal}\left(C_{metal}+C_{MIM}\right)\right]}{\left[\left(R_{metal}+R_{poly}\right)\left(C_{metal}+C_{MIM}\right)\right]}\\ =\frac{\left(N_{DC,1}-q_{1}\right)\left(N_{DC,2}-q_{2}\right)}{\left(N_{DC,3}-q_{3}\right)}\frac{T_{CLK}}{\ln(2)}\\ =\frac{\left(N_{DC,1}N_{DC,2}-N_{DC,2}q_{1}-N_{DC,1}q_{2}+q_{1}q_{2}\right)}{\left(N_{DC,3}-q_{3}\right)}\frac{T_{CLK}}{\ln(2)} \end{array} \end{array}$$

Since $q_{n}$ (where $q_{n}\in \left(0,1\right]$) is much smaller than $N_{DC,n}$, the Equation (A1) can be approximated as Equation (15).
